# Factors influencing FDA drug approval decisions beyond clinical trial evidence: a scoping review

**DOI:** 10.1093/haschl/qxag201

**Published:** 2026-08-04

**Authors:** Robin Forrest, Martin Lodge, Ameet Sarpatwari, Huseyin Naci

**Affiliations:** Department of Health Policy, The London School of Economics, London WC2A 2AE, United Kingdom; Department of Government and Centre for Analysis of Risk and Regulation, The London School of Economics, London WC2A 2AE, United Kingdom; Harvard Pilgrim Health Care Institute and Harvard Medical School, Boston, United States; Department of Health Policy, The London School of Economics, London WC2A 2AE, United Kingdom

**Keywords:** FDA, US Food and Drug Administration, drug approval process, pharmaceutical policy, regulation, regulatory agencies

## Abstract

**Introduction:**

The Food and Drug Administration's (FDA) approval decisions are expected to rest primarily on clinical evidence, yet concerns persist about whether political pressure, commercial interests, or institutional dynamics also influence the approval process.

**Methods:**

We conducted a scoping review of empirical studies published between 1990 and 2025 evaluating factors other than clinical evidence in FDA drug approval decisions, following the Preferred Reporting Items for Systematic Reviews and Meta-Analyses extension for Scoping Reviews (PRISMA-ScR) framework.

**Results:**

Fifteen studies examined 5 categories of factors: firm-level attributes, FDA organizational structures, procedural factors, regulatory reforms, and external pressures including media attention and political salience. Most studies (10/15) evaluated these factors using speed of FDA drug approval as a primary outcome, rather than the decision to approve or reject, or a measure of decision quality. Findings were mixed, with the most consistent evidence for close agreement between advisory committee recommendations and approval decisions, identified across independent research teams. More limited evidence also suggested external stakeholder pressure may affect review times.

**Conclusion:**

A paucity of empirical evidence likely points to the inherent difficulty of demonstrating influence on agency decision-making. More research is needed to evaluate the potential influence of factors beyond clinical evidence on approval decisions, and the extent to which these decisions ultimately benefit patients.

## Introduction

A central mandate of the US Food and Drug Administration (FDA) is to protect public health by ensuring that the benefits of new drugs outweigh their risks.^1,2^ This role carries global significance, as FDA approval decisions shape evidentiary standards followed by regulators worldwide. In principle, approval decisions would be expected to rest primarily on evidence of clinical efficacy and safety, consistent with the FDA's technical expertise and privileged access to clinical trial evidence. However, a series of controversial approvals over the past 2 decades has raised concerns that FDA decisions may be unduly influenced by factors other than clinical evidence. These include pressure from the executive branch and Congress,^3^ internal FDA politics and disagreements between scientific reviewers and senior agency leadership,^4^ intense patient group advocacy, and close relationships between FDA and industry, among others. If supported by empirical evidence, these concerns risk undermining the legitimacy of the FDA drug approval process and the agency's ability to fulfill its public health mandate.

Concerns about FDA decision-making have recurred over time, brought into focus by several high-profile approvals. In 2016, the FDA approved eteplirsen for Duchenne muscular dystrophy despite a negative advisory committee vote and amid strong patient advocacy, after the Center Director overruled her scientific review team, having cited concern that rejection could threaten the manufacturer's financial viability.^5^ In 2021, the FDA approved aducanumab for Alzheimer's disease against the recommendation of an independent FDA advisory committee, which had cited substantial efficacy and safety concerns.^6-9^ It drew strong criticism,^10-13^ and prompted a Congressional inquiry that found the approval followed an “atypical” and “substantially abbreviated review process” involving inappropriate collaboration between the FDA and Sponsor.^14^ More broadly, concerns have been raised about evidentiary standards applying to FDA's use of the accelerated approval pathway.^15^ Together, such cases have fueled debates about FDA decision-making and contributed to a documented decline in public trust in the agency,^16,17^ which is essential for its mandate to be effective.^18^

In practice, FDA approval decisions are rarely straightforward, requiring scientific and value judgments under uncertainty, and a balance between competing pressures.^3^ The agency must balance unmet needs against upholding evidentiary standards, and manage societal expectations for timely access to new treatments.^19,20^ Consequently, the speed of drug approval has itself become a highly politicized issue, an important dimension of the approval process, and a major focus of both reform and empirical study.^21,22^ Like other government agencies, the FDA must also navigate institutional factors including funding arrangements, statutory deadlines, and performance targets.^21^ Meanwhile, sponsors have strong incentives to secure favorable and timely approvals, generating strong political pressure for access to new drugs. Although the FDA is skilled at balancing these competing pressures,^15^ using its discretionary authority to weigh them alongside the clinical evidence,^23^ the absence of explicit decision-making criteria makes it difficult to distinguish undue influence from legitimate regulatory discretion, particularly when decisions are later contested.

A substantial body of research has examined the quality and quantity of clinical evidence supporting FDA approvals,^24-26^ but comparatively little is known about the extent to which factors beyond clinical data influence FDA decision-making. Several theoretical frameworks from across the social sciences may help explain how and why agencies may be influenced to act in ways that extend beyond, or conflict with, their primary mandate.^27^

Public interest theories of regulation suggest that regulators act as neutral, expert arbiters, guided solely by evidence and the pursuit of public welfare.^28^ This is broadly consistent with the expectation of the FDA to make decisions that protect public health, guided by clinical evidence, while balancing additional clinical contexts such as unmet medical need or treatment urgency. However, this view is often regarded as incomplete, as it overlooks that politicians pursue electoral interests, regulated industries have strong incentives to influence those overseeing them, and other organized stakeholders compete to shape regulatory outcomes.^27^

By contrast, the economic theory of regulation,^29^ alongside interest group and regulatory capture theories,^30^ suggests that regulation is shaped by the organized interests of those it oversees.^31^ This may be particularly salient in the pharmaceutical context, given the FDA oversees the largest global market for pharmaceuticals. Such influence may operate through several channels: the revolving door between industry and senior FDA officials,^32-34^ congressional lobbying of the FDA,^35^ direct advocacy by industry-sponsored patient groups,^36^ and other mechanisms that are challenging to observe and quantify.

Beyond external pressures, bureaucratic and institutional theories draw attention to forces within the agency itself. For example, Carpenter^37^ suggests the FDA's decisions are shaped not only by clinical evidence but by reputational concerns, that is, the agency's desire to maintain status among scientific, political, and public audiences. Other frameworks across disciplines that explain agency actions and regulatory outcomes can also be applied to pharmaceutical regulation.^27,38,39^

Despite the salience of this issue, the extent to which existing concerns are supported by empirical evidence remains unclear. This scholarship is often conceptual, spans multiple disciplines, and uses varying analytic frameworks, making it hard to draw conclusions beyond individual approvals. A multidisciplinary synthesis is needed to identify common approaches, clarify where empirical findings converge or diverge, and highlight priorities for future research to better understand factors that may influence FDA drug approval decisions.

This study undertakes a multidisciplinary review to identify empirical studies evaluating the potential influence of factors beyond clinical trial data on FDA drug approval decisions. Specifically, we: (1) identify which nonclinical factors have been empirically investigated; (2) synthesize the evidence, highlighting where findings converge or diverge; and (3) characterize the evidence base (disciplines, origins, and empirical approaches) and identify key gaps to guide future research.

## Methods

We conducted a scoping review following Preferred Reporting Items for Systematic Reviews and Meta-Analyses extension for Scoping Reviews (PRISMA-ScR) guidance^40^ to identify empirical studies evaluating the influence of factors beyond clinical trial data on FDA drug approval decisions. A scoping review was chosen because it was well suited to the broad aims of our study: to summarize empirical evidence and map key concepts across multiple fields of literature.^41,42^

### Scope and eligibility criteria

To be eligible, studies must have met 5 inclusion criteria. (1) Focus on FDA drug approval decisions, including their speed. We restricted the review to approvals because they represent a discrete, high-stakes regulatory decision with relatively consistent stakeholders, incentives, and evidentiary standards. This enabled comparison of factors across studies that would not be comparable across decision types arising in different contexts, and resting on different evidentiary bases. Other drug-related actions (eg, label changes or safety withdrawals) were not eligible. Both the approval decision (ie, to approve or reject) and approval speed were eligible outcomes, as they reflect important aspects of the same regulatory process, with factors influencing one considered likely to be relevant to the other. (2) Empirical studies, defined as research generating data-driven findings from observed or measured phenomena. Qualitative, quantitative, and mixed-methods studies were all potentially eligible. We excluded commentaries and work relying solely on theory or opinion. (3) Examination of at least one factor (explanatory variable) capturing a potential influence beyond clinical trial data, since trends in the quality and quantity of clinical evidence underpinning FDA approval decisions have been well characterized previously.^24,25,43,44^ (4) Authors must have explicitly connected their findings to FDA approval decisions, in the form of at least one hypothesis, theory, or conclusion. It was not sufficient for a study to empirically evaluate a factor that could plausibly influence the FDA. Our aim was to synthesize theories already tested and explicitly linked to FDA decision-making, not to infer new mechanisms from others' empirical work. (5) Peer-reviewed articles, or book chapters and books if findings were original and not duplicated elsewhere. Reports, news articles, and commentaries were excluded. Full eligibility criteria are provided in Table 1.

**Table 1.** Inclusion and exclusion criteria.

Topic	Inclusion criteria	Exclusion criteria
Focus and/or relevance	Included studies must focus primarily on the FDA, and authors must explicitly relate study findings to FDA decision-making processes.	Studies focused on other regulatory agencies. Studies that do not offer at least one hypothesis, theory, or conclusion relating to FDA decision-making.
Study type	Empirical studies utilizing direct observation, experimentation, or measurable evidence in relation to a specific research question or hypothesis, including quantitative studies, empirical case studies, interviews, document analysis, database analysis, etc.	Nonempirical studies.
Explanatory variables	Studies that examined at least one explanatory variable capturing a potential influence beyond clinical trial data (eg, firm size)	Studies examining the influence of clinical trial data (quality or quantity) on approval outcomes.
Outcomes	FDA drug approval decisions (ie, positive/negative decisions, speed of decisions, etc.)	Studies focusing on other regulatory outcomes (eg, withdrawals). Studies investigating regulation of medical devices, food, tobacco, alcohol, consumer products, marketing or advertising.
Publication type	Peer-reviewed articles. Books and book chapters may be included if findings are original and not duplicated in a peer-reviewed article.	Reports, News Articles, and Commentaries

### Search strategy

We used an iterative, seed-based approach to develop and refine search terms, given the exploratory aim of this review. First, we began with 18 seed articles identified in the literature that considered FDA approval decision-making and the broader politics of FDA drug review. We then mapped key concepts and subject headings (eg, MeSH terms) from each article. Second, in consultation with a political scientist with expertise in regulatory governance and administration, we searched for potentially relevant concepts (ie, influence) related to broader regulatory agency decision-making. Together, these approaches yielded 3 core concepts: the FDA, drug approval decisions, and factors that may influence the decision-making of regulatory agencies (excluding clinical data). In consultation with a research librarian, we refined these search terms and combined core concepts until a single search identified all seed articles.

To capture relevant literature across disciplines, we searched MEDLINE (via Ovid) and Scopus, adapting search syntax accordingly. These databases were selected for their comprehensive coverage of biomedical, regulatory, and multidisciplinary research relevant to health, regulation, and the social sciences. Studies were limited to 1990 onward and English language. Full search terms are provided in Supplementary Appendix A.

To illustrate the range of disciplines and empirical approaches considered, and the boundaries of the review's scope, some examples of screened studies that did not meet the inclusion criteria are noted in Supplementary Appendix B alongside their reasons for exclusion.

### Supplementary searches

To supplement the systematic search, we conducted backward citation searching by screening the reference lists of all included studies. References deemed potentially relevant were retrieved as full-text articles for eligibility assessment. We also conducted forward citation searching of included studies to identify subsequently published work citing them, using the Scopus “Cited Reference Search” tool, where studies were indexed, and Google Scholar. Finally, we used ResearchRabbit to identify additional studies closely networked with the included references.

### Screening

Screening and application of eligibility criteria were conducted by a single reviewer. A second reviewer independently screened a random sample of titles/abstracts and articles assessed at full-text screening. Raw percentage agreement was calculated at each stage. Unclear cases were flagged during initial screening, carried forward for full-text screening, and discussed between authors where necessary.

### Data charting, summarizing, and reporting findings

From each included study, we recorded the author, year, main study questions, study type (analytical approach), study period, relevant explanatory variables, outcome variables, key findings, and the authors' interpretation of the evidence (ie, the extent to which the explanatory variables may influence FDA decision-making, and why). From these, we grouped factors thematically into categories, informed by the broader public administration and health policy literature.^38^

## Results

### Search results

**Figure 1.**
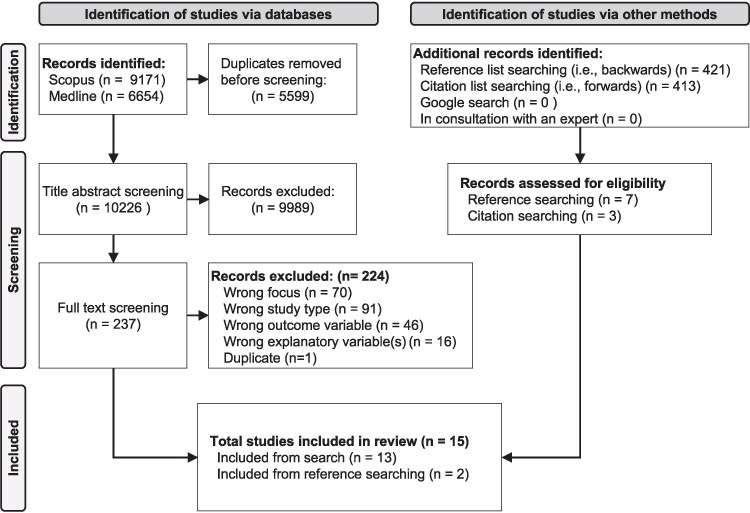
PRISMA-ScR flowchart. Abbreviation: PRISMA-ScR, Preferred Reporting Items for Systematic Reviews and Meta-Analyses extension for Scoping Reviews.

MEDLINE (via Ovid) and Scopus were searched in February 2025. The search identified 10 226 unique records, of which 9989 were excluded at title and abstract screening. Full-text review of the remaining 237 records identified 13 eligible studies. Supplementary searches identified 7 potentially eligible studies, of which 2 met inclusion criteria. In total, 15 empirical studies were included (Figure 1). Independent second-reviewer screening of a random sample (350 titles/abstracts; 50 of the 237 full-text articles, 21%) showed high agreement: 98% (343/350) at title/abstract and 100% (50/50) at full text, with both reviewers independently identifying the same 3 eligible studies within the sample. All title-abstract discordances involved records included by the primary reviewer alone that were subsequently excluded at full text.

### Overview of study disciplines, empirical approaches, and origins

The included studies span several academic disciplines: 9 from the political science and public administration literature,^37,45-52^ 1 from the strategic management literature,^53^ and 5 from the health policy literature^54-58^ (Table 2). Most studies employed quantitative methods, especially multivariable or survival regression models. Alternative approaches included retrospective analyses of advisory committee votes,^56,57^ and case-based process tracing with archival data.^37^ One study used qualitative interviews with regulators to compare institutional cultures.^54^ The evidence base is also concentrated by authorship: over half of the 15 studies derive from 2 research programs, with 5 led by Carpenter^,37,47,48,50,52^ and 3 by Olson^45,46,49^. This concentration is most pronounced among earlier studies, whereas more recent work stems from a wider range of teams.

**Table 2.** Characteristics of included studies.

Author (year) title	Study discipline	Study type and study period	Explanatory variable(s) (relevant only)	Dependent variable(s)/outcomes (main only)	Key findings (main/relevant only)	Proposed interpretation of findings (main/relevant only to informal decision-making)
**Olson (1997)** ^45^ Firm characteristics and the speed of FDA approval.	Political science, public administration	Retrospective study using multivariable regression (1990-1992)	R&D intensity of firm No. of concurrent drug applications Domestic (US) vs foreign Firm size Therapeutic novelty	FDA review time	Larger, research-intensive, less diversified, foreign (vs domestic) firms and those with more submissions benefit from faster approval	Firm characteristics and reputation signals quality and reduces uncertainty for FDA leading to faster review time A firms' experience builds trust and familiarity with the FDA Larger firms have more political clout and regulators may be more responsive to larger firms Capture theory cannot explain why some regulators are more responsive to different firms in the same industry
**Olson (2000)** ^46^ Regulatory reform and bureaucratic responsiveness to firms: the impact of user fees in the FDA.	Political science, public administration	Multivariable regression analysis (1990-1995)	Firm characteristics (R&D intensity, size, experience, domestic vs foreign) Drug characteristics (priority status, therapeutic class)	FDA review time	User fees significantly reduced approval times FDA became less responsive to firm characteristics after introduction of user fees Greatest impact on speed of approval for novel drugs	User fees create a financial incentive for FDA to process more approvals and induce change in FDA behavior User fees substitute some of the prior impact that firms' characteristics may have had on approval times, leading to better equity in approval processes User fees could lead FDA reviewers to accept more Type I risk^a^ in exchange for reduced Type II errors^b^
**Carpenter (2002)** ^47^ Groups, the media, agency waiting costs, and FDA drug approval.	Political science, public administration	Optimal stopping model (1977-2000)	Wealth of advocacy groups No. of advocacy groups Media coverage Congressional attention Firm experience and clout Order of market entry	FDA review time	Advocacy group wealth and media coverage reduce FDA review times (particularly when political salience is high) But, many smaller groups may reduce influence due to coordination problems External politics influences FDA approval more through media and disease salience than through elected officials	There is a substantial political cost to the FDA of delay in approval which increases with salience FDA balances political cost with risking its reputation, maintaining a focus on reducing type I errors Media and actions of advocacy group increase salience, and increase waiting costs for FDA (AIDS drugs being an illustrative example)
**Carpenter et al. (2003)** ^48^ Approval times for new drugs—Does the source of funding for FDA staff matter?	Health policy, political science	Multivariable survival analysis (1977-2000)	FDA staffing levels Firm characteristics (sales, lobbying, past NDA submissions) Disease characteristics Priority status	FDA review time	Approval times decreased by 3.3 months for every additional 100 FDA staff No evidence larger or more politically active firms benefited more from PDUFA	Increased staffing, rather than source of funding (ie, industry), led to faster approval times post-PDUFA enactment (which began 5 before PDUFA) Nearly all reduction in approval times could have been achieved through redirection of FDA funds
**Olson (2004)** ^49^ Managing delegation in the FDA: reducing delay in new-drug review.	Public administration, political science	Multivariable regression and survival analysis (1971-1998)	FDA budget/CDER staffing Current approval workload at FDA Firm characteristics (R&D intensity, size, experience, etc.) Drug characteristics	FDA review time	Implementation of user fees led to a 34% reduction in mean drug review time, independent of resource increases FDA became less responsive to firm characteristics (eg, R&D intensity), indicating more equitable review Review times dropped most for novel drugs and for therapeutic classes historically associated with delay (eg, CNS, cardiovascular)	Analyses impact of user fees, controlling for changes in resource and workload over time Additional FDA resources alone cannot explain reduction in drug-review times FDA more sensitive to reducing delays User fees make FDA dependent on industry for some of its funding which could lead to collusion between the industry and FDA (and potentially capture given all firms see increase in speed of approval) If benefits of faster approvals outweigh any new risks, then capture is negated
**Carpenter (2010)** ^37^ Reputation and power: organizational image and pharmaceutical regulation at the FDA.	Political science/public administration	Book-length empirical analysis using mixed methods combining empirical case studies, archival research, interviews, process tracing, and other analysis.	External political pressure (eg, Congress, media) Internal scientific expertise and structure Stakeholder expectations (industry, scientific community, patient groups) Use of advisory committees and public visibility	Drug approval decisions (eg, timing, outcomes) Use of advisory committees Regulatory response to crises Post-market enforcement behavior	The FDA's decisions are shaped to maintain reputation for scientific rigor, neutrality, and public protection across multiple audiences Reputational concerns make the FDA a cautious regulator (sometimes slow), particularly in high-risk or high-profile cases The FDA uses tools like advisory committees, public hearings, and selective transparency to maintain credibility and defend against external political pressures	FDA actions and decision-making are shaped by how it is perceived by external audiences The FDA's authority is reputational as well as legal FDA actions balance the expectations of multiple, often conflicting, stakeholders The FDA delays or denies approval when risk is high to protect its legitimacy and reputation FDA authority is reinforced through alignment with scientific and medical elites
**Carpenter et al. (2010)** ^50^ Early entrant protection in approval regulation: theory and evidence from FDA drug review.	Political science	Multivariable survival analysis and other statistical methods (1950-2006)	Order of entry into therapeutic class Firm attributes (size, submissions, experience) Disease characteristics (advocacy groups, media attention, prevalence) Policy developments (1962 amendments and PDUFA)	FDA approval time	FDA review times increase with order of entry: first to market receive fastest FDA review Positive association between order of market entry for a drug and its approval time. Advocacy/salience explain variation in this gradient whereas firm-level factors do not The pattern intensified after the 1962 Kefauver-Harris Amendments, but was not significantly affected by PDUFA	Consumer co-optation whereby FDA prioritizes early entrants to defuse political pressure from organized patients FDA delays approvals unless the political benefit of acting outweighs reputational risk Evidence against capture as firm size, sales, and submission history do not significantly condition early entrant protection Politics/salience (ie, advocacy group strength and media attention) shape entry-order/approval time gradient
**Lavertu and Weimer (2011)** ^51^ Federal advisory committees, policy expertise, and the approval of drugs and medical devices at the FDA.	Public administration/political science	Multivariable regression, survival analysis and other statistical methods (1997-2006)	Proportion of approval votes among committee members Interest group presence (advocacy) and media salience. NME Designation status (priority review, Accelerated Approval, expedited review, orphan) Presidential administration	FDA decision to consult advisory committee FDA approval decision (yes/no) FDA review time (both from application and from committee meeting)	Stronger committee support increases the likelihood and speed of FDA approval after controlling for other covariates FDA often seeks committee advice when uncertainty is high (NMEs), when the political stakes are higher, and when in-house capacity is limited Committee consultation does not increase review times for drugs and can reduce time-to-approval post-meeting Political party in power did not influence how FDA considers committee advice	Advisory committees are used by the FDA to reduce epistemic and political uncertainty Committees provide legitimacy and “political cover” for the FDA Advisory committee support (especially consensus) reduces review time, suggesting that FDA moves more quickly when external validation reduces uncertainty Measures of interest group activity and disease salience predict likelihood of committee use and influence timing of FDA approval decisions
**Carpenter et al. (2012)** ^52^ The complications of controlling agency time discretion: FDA review deadlines and postmarket drug safety.	Political science/public administration	Multivariable survival analysis (1950-2008)	Approval within 2 months of deadline Priority status Firm's regulatory experience Drug novelty Submission year	Timing of FDA approval Postmarket safety events, including (black box warnings, safety alerts, safety-based withdrawals and dosage-form discontinuations)	FDA approvals pile up just before review deadlines, especially after PDUFA implementation Drugs approved in the 2 months before a deadline are more likely to receive black-box warnings, safety withdrawals and safety alerts after approval Decision timing constraints imposed by deadlines may impair FDA's procedural diligence and compromise regulatory quality	Deadlines are powerful administrative tools that aim to improve agency efficiency and timeliness, signaling accountability and better oversight/responsiveness of the FDA. FDA adjusts behavior to meet deadlines In practice, deadlines can reduce quality of decisions and lead to patient harm
**Kim (2012)** ^53^ Arbiter of science: institutionalization and status effects in FDA drug review 1990-2004.	Management/political science	Multivariable survival analysis (1990-2004)	Patent citations Innovation (NMEs) Advisory committee use Entry order (as proxy for unmet need) Recent product withdrawals	FDA review time	Firms with higher scientific status receive faster FDA approval after controlling for drug quality, firm size, political contributions, and experience. Status effects are stronger when uncertainty is high (eg, NMEs, use of advisory committees, new disease categories)	The FDA has adopted scientific values as central to its legitimacy. The FDA looks at signals of a firm's scientific stature to resolve some uncertainty A firm's status (in this case scientific status) provides external signals the FDA considers when evaluating new drugs Approving a drug from a high-status firm involves lower reputational risk for the FDA
**Tafuri et al. (2014)** ^54^ How do the EMA and FDA decide which anticancer drugs make it to the market? A comparative qualitative study on decision makers' views.	Health policy, regulatory science	Qualitative, cross-sectional interview study with 13 (6 FDA) senior drug regulators (2012)	Patient/stakeholder input Interactions with industry Public hearings Internal organization Risk attitudes	FDA approval decisions Benefit-risk evaluations	FDA (and EMA) prioritize efficacy, but diverge in how they define and weigh evidence FDA is more risk-tolerant, more willing to approve drugs based on activity or surrogate endpoints, and more receptive to patient input EMA is more cautious, and more skeptical of public hearings and patient advocacy Differences in decisions stem from cultural, structural, and institutional norms, not just clinical data	FDA's culture encourages faster access and more risk tolerance even under uncertainty FDA's integration of patients, advisory committees, and public hearings affects both transparency and regulatory outcomes Impact of structural organization on decision-making ie, FDA relies on discipline-specific teams FDA is more centralized and open to innovation and dialogue whereas EMA is more influenced by national systems and bureaucratic procedures
**Tibau et al. (2016)** ^55^ Oncologic Drugs Advisory Committee (ODAC) recommendations and approval of cancer drugs by the US Food and Drug Administration.	Health policy, regulatory science	Retrospective observational study of 82 ODAC meetings and subsequent FDA approvals (2000-2014)	FCOIs (presence, type, sponsor vs competitor) Application type (new vs supplemental, regular vs accelerated) Other formal factors (trial design, etc.)	FDA approval decision ODAC recommendation (vote)	ODAC recommendations and FDA approvals strongly aligned No statistically significant link between FCOIs and FDA approval (or ODAC votes) FCOIs among ODAC members declined over time. Other formal factors linked to approval ie, use of multiple trials, randomization, trial design etc.	FDA advisory committee recommendations have a strong effect on final approval outcomes, despite being advisory in principle FDA readily uses flexible evidentiary standards and applies them in a context-dependent way, ie, especially in rare diseases Over time, the FDA reduced FCOIs among ODAC members, reflecting institutional concerns about public trust and bias
**McCormack (2018)** ^56^ United States Food and Drug Administration Advisory Committee outcomes and agency approval analysis from 2010 to 2015.	Health policy, regulatory science	Retrospective observational study of FDA CDER advisory committee meetings (2010-2015)	Advisory committee vote (yes/no) Type of voting question (eg, approval, safety, efficacy) Year of meeting	FDA approval decision Concordance between vote and FDA decision Reasons for disagreement	FDA actions mostly align with ACs When disagreement occurred, it was usually when FDA did not approve a product despite AC support (81% of disagreements) Reasons for disagreement included were mostly clinical/evidence focused or unknown (24%). The study confirms that AC recommendations are highly influential but not determinative	FDA advisory committees are influential but not binding FDA discretion reflects institutional norms and expertise on areas outside remit of advisory committees FDA can choose to go against recommendations (rarely), and when it chooses to do so is mostly more cautious
**Zhang et al. (2019)** ^57^ Association between Food and Drug Administration Advisory Committee recommendations and agency actions, 2008-2015.	Health policy, regulatory science	Retrospective observational study of 376 FDA advisory committee meetings with dichotomous votes (2008-2015)	Action type (eg, approval, etc.) Committee consensus level Public speaker favorability Conflicts of interest Product orphan or expedited designation Media coverage	Concordance or discordance between the advisory committee recommendation FDA's final action (including approval)	FDA actions mostly align with ACs In discordant cases, FDA was more likely to take a more cautious position than the committee, especially for approvals No association between discordance and conflicts of interest, media attention, or public speaker favorability	FDA may act more conservatively than its advisory committees due to reputational risk aversion, ie, to avoid future regulatory reversals, public criticism, etc. FDA decision-making reflects a participatory bureaucracy in which the agency ultimately relies on its own scientific expertise and institutional mandate FDA's ability to diverge from advisory committee recommendations illustrates its institutional independence allowing it to prioritize long-term credibility and regulatory consistency over external alignment
**Daval et al. (2023)** ^58^ Association of advisory committee votes with US Food and Drug Administration decision-making on prescription drugs, 2010-2021.	Health policy, regulatory science	Retrospective observational study of 409 advisory committee meetings (2010-2021)	AC vote (ie, positive/negative) Vote type (initial approval, etc.) Time since vote	FDA decision to approve/reject/other action taken Alignment or discordance with AC vote	FDA actions mostly align with ACs Alignment highest for positive votes The FDA approved drugs more often than ACs recommended, particularly after negative votes The FDA declined to reconvene advisors before most approvals of drugs that had previously received a negative vote FDA sought independent expert advice less frequently over time	FDA values advisory committee input but retains the final say, increasingly to approve drugs even after negative votes FDA uses advisory committees to confer public legitimacy but avoids reconvening them before controversial reversals to manage reputational risk and avoid external scrutiny. Decline in advisory committees suggests FDA is strategic in when and how it seeks external input

^a^A Type I error in drug regulation occurs when a drug is approved despite lacking true efficacy or safety, ie, a false positive decision. ^b^A Type II error occurs when a drug that is effective or safe is not approved, ie, a false negative decision.

Abbreviations: AC, advisory committee; AIDS, acquired immunodeficiency syndrome; CDER, Center for Drug Evaluation and Research; CNS, central nervous system; EMA, European Medicines Agency; FCOI, financial conflict of interest; FDA, Food and Drug Administration; NDA, New Drug Application; NME, new molecular entity; ODAC, Oncologic Drugs Advisory Committee; PDUFA, Prescription Drug User Fee Act; R&D, research and development.

### Outcome variables

Ten studies used review speed (eg, review time or time to approval) as the primary outcome. These included all studies from the public administration and political science literature identified in the review, and all studies published before 2014. The remaining studies, by Tafuri et al.,^54^ Tibau et al.,^55^ McCormack,^56^ Zhang et al.,^57^ and Daval et al.^58^ focused on approval outcomes (ie, whether an application was approved or rejected) and the factors associated with those outcomes. Although these are 2 dimensions of the same regulatory process, factors in each study were typically evaluated against only one—Lavertu and Weimer^51^ being the exception, evaluating both outcomes. A finding about review speed does not necessarily extend to an approval decision, or vice versa. We therefore discuss factors evaluated against each outcome separately.

### Factors influencing FDA drug approval decisions (explanatory variables)

**Figure 2.**
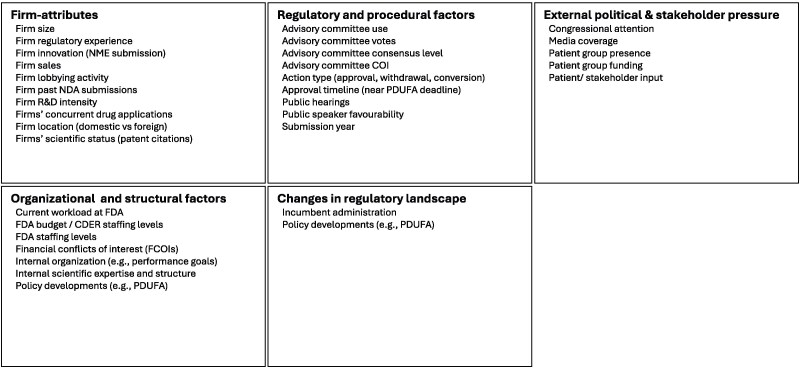
Overview of factors evaluated in the empirical literature for influence on FDA drug approval decisions. The terminology used to classify factors in this figure is intended to group similar bodies of research and may not align precisely with how these terms are used in other literature. Abbreviations: CDER, Center for Drug Evaluation and Research; COI, conflict of interest; FCOI, financial conflict of interest; FDA, Food and Drug Administration; NDA, New Drug Application; NME, new molecular entity; PDUFA, Prescription Drug User Fee Act; R&D, research and development.

Five types of factors beyond clinical trial data were evaluated in the empirical literature (Figure 2). (1) Firm-level attributes refer to characteristics of the sponsor. (2) Organizational or structural factors refer to internal features of the agency, such as staff levels, workload and internal resources. (3) Procedural factors refer to features of the decision-making process for individual approvals, such as the consultation of advisory committees. (4) Changes in the regulatory landscape refer to external policy changes. (5) External political pressures include factors such as congressional and political salience, and patient or disease-advocacy group activity.

#### FDA responsiveness to firm-level attributes and expertise

Several studies investigated the potential influence of firm-level attributes on the speed of FDA drug review, drawing mixed conclusions. Olson^45^ found that larger firms, those with greater R&D intensity, and those with more prior submissions had faster review times, suggesting that a firm's characteristics and reputation signal information to reviewers about application quality. Extending this analysis, Olson^46,49^ found that this diminished post-Prescription Drug User Fee Act (PDUFA), suggesting the FDA became less responsive to these firm-level attributes after implementation of the new user-fee system. Carpenter et al.^37,47,48,50^ found that firm characteristics had little to no influence on FDA approval times for new drugs. Kim^53^ found that drugs from high-status firms were approved faster, suggesting the FDA relies on external signals of quality such as status to resolve uncertainty during drug approval.

#### Influence of FDA's organization and structure

Several studies investigated the potential influence of FDA's internal organization and structure on the speed of FDA drug review. Carpenter et al.^48,52^ and Olson^49^ evaluated the potential influence of organizational structures and resource allocation within the FDA on review times. Carpenter et al.^48^ found that an increase in staffing, rather than the source of funding, led to faster review times post-PDUFA (1992) and found no evidence to suggest that larger or more politically active firms benefited more from PDUFA. Olson^49^ found that higher agency workload was associated with longer drug review times, suggesting that workload pressure influenced review speed, even after an increase in agency resources (post-PDUFA). Uniquely among the identified studies, Tafuri et al.^54^ used in-depth interviews with FDA and EMA (European Medicines Agency) regulators to examine why the 2 agencies sometimes reach divergent approval decisions. Aside from viewing clinical endpoints differently, regulators attributed divergence partly to institutional and cultural differences, namely the FDA's greater willingness to take risks to speed up access, and partly to differences in how the 2 agencies interact with external stakeholders (discussed below).

#### Influence of procedural factors

Procedural factors were evaluated against review speed and approval decisions, in some cases both within a single study. Carpenter et al.^52^ found that after implementation of the 1992 PDUFA, approval decisions clustered just before regulatory deadlines and were associated with more postmarket safety issues, suggesting a potential link between review deadlines and both the timing and quality of approval decisions. Several studies evaluated advisory committee use, and these differed in the outcome they examined. Lavertu and Weimer^51^ estimated the influence of committee votes on approval decisions and speed, modeling the approval decision, the time to approval, and the agency's prior decision of whether to consult a committee. They found that an increase in the proportion of committee members voting for drug approval increased the probability of FDA approval and (in a separate model) reduced approval time. Tibau et al.,^55^ McCormack,^56^ Zhang et al.,^57^ and Daval et al.^58^ evaluated the degree of agreement between committee recommendations and approval decisions, and the factors associated with discordance. Advisory committee recommendations strongly correlated with FDA decisions, although the agency occasionally diverged, usually when it adopted a more cautious stance. Authors suggested that advisory committees may help reduce uncertainty and offer greater legitimacy or political cover, particularly for controversial decisions. Zhang et al.^57^ found that conflicts of interest among committee members, public testimony, and media coverage were not associated with whether the FDA agreed or disagreed with advisory committee recommendations. Regulators interviewed in the Tafuri et al.^54^ study attributed divergence between FDA and EMA decisions to differences in how the 2 agencies engage external stakeholders, including patients and pharmaceutical companies. The FDA's process was characterized as involving closer collaboration with industry from the early stages of drug development and involved greater use of public hearings at which patient representatives can be voting members.

#### Influence of shifts in regulatory landscape

Several studies assessed the potential influence of changes in the regulatory landscape and legislation surrounding approval on the speed of drug review. Olson^46,49^ found that implementation of user fees led to shorter review times and diminished the impact of firm-level characteristics on review times. Carpenter et al.^48^ showed that staffing increases under PDUFA improved performance, while Carpenter et al.^50^ further demonstrated that the post-1962 regulatory environment intensified early-mover advantages in review speed.

#### Influence of external political pressures and stakeholders

Multiple studies evaluated the potential influence of external political and stakeholder pressure on approval speed and approval decisions. Carpenter^47^ showed that media attention, advocacy group wealth, and disease salience were associated with faster approval times, which Carpenter attributed to increased “waiting costs” of regulatory delay. Carpenter et al.^50^ extended this analysis with the theory of consumer co-optation, arguing that early entrant drugs receive faster reviews because the FDA seeks to diffuse patient advocacy pressure. Lavertu and Weimer^51^ and Zhang et al.^57^ similarly noted that advocacy salience and political sensitivity were associated with a higher likelihood of advisory committee use. Carpenter^37^ offered the most comprehensive theoretical model with a reputational governance theory suggesting that the FDA makes decisions not only to follow science or law but to maintain legitimacy among multiple audiences: Congress, industry, media, and the public. Carpenter argued that the FDA is especially cautious about approving drugs where reputational damage from approval of a harmful drug would be severe, making it particularly responsive to high-profile political and public scrutiny.

## Discussion

FDA approval decisions have, over time, raised concerns that political pressure, institutional constraints, commercial interests, and other factors beyond clinical evidence may unduly shape regulatory outcomes. However, the extent to which these concerns are supported by empirical evidence remains unclear. We conducted a scoping review to identify empirical studies evaluating the influence of factors beyond clinical trial data on FDA drug approval decisions.

### Limited empirical evidence

Despite frequent claims that political pressures influence FDA approval decisions, we identified only 15 studies testing these claims empirically. One explanation could be that informal influence is inherently difficult to measure because it operates through indirect channels that do not lend themselves to empirical testing.^31^ Another is likely data availability and limited transparency of decision-making processes at the FDA. The included studies also represent a small set of authors that dominate this empirical field. Over half of the eligible studies stem from 2 research programs, led by Carpenter (5 studies) and Olson (3 studies). As studies within these research programs at times shared drug approval cohorts, time windows, and theoretical commitments, agreement within them may carry less evidential weight than consistency across independent teams. Conclusions across studies should therefore be read as reflecting a small set of research programs rather than a diverse body of established scholarship.

### Evidence of influence

Findings were most consistent for advisory committee use (studied mainly against approval decisions) and external stakeholder pressures (studied against review speed). While the FDA states that advisory committees exist to provide independent expert input and “help make sound decisions,”^59^ authors suggested their role extends beyond scientific guidance to reduce regulatory uncertainty and provide political legitimacy. Several studies, conducted by independent research teams, found that advisory committee recommendations closely aligned with final FDA decisions. Such concordance may be expected, and close agreement may arise without any causal effect of committee votes on final decisions. Only one study was designed to estimate the influence of committee votes rather than to document agreement, finding that a higher proportion of committee members voting for approval increased the probability of drug approval and reduced time to approval.^51^ When discordance from committee votes does occur,^59^ it can carry significant consequences, as seen in recent controversial approval decisions.^5,14^ A recent study found that the number of new approved drugs receiving advisory committee review decreased from 59% in 2011 to only 6% in 2021.^60^ This decline may reflect strategic limitation of independent scrutiny rather than passive disengagement, raising questions about whether the FDA selectively deploys advisory committees to manage reputational risk.^61^ To the extent that the FDA selectively deploys advisory committees, the observed agreement between committees and agency decisions in the identified literature may be partly artifactual. Additional research is needed to evaluate the causal effect of committee votes on FDA decisions.

Evidence from several of the included studies supported the theory that heightened media coverage, advocacy group activity, and political salience have historically led to accelerated review times. Carpenter's empirical work, for example, showed that media attention, advocacy group wealth, and disease salience were each associated with faster approvals.^47^ Further independent research testing the influence of these factors on approval outcomes would strengthen these findings.

Through interviews with FDA and EMA regulators, Tafuri et al.^54^ identified 3 sources of divergence between the agencies: differing interpretations of clinical endpoints (consistent with prior work),^62,63^ the FDA's greater willingness to accept risk, and differences in how the agencies engage industry and patients. The fact that regulators themselves attributed divergence to institutional culture and stakeholder engagement suggests factors beyond clinical evidence may influence approval. As a small qualitative study, these findings warrant confirmation by larger quantitative work.

For several frequently scrutinized factors, we found no empirical evidence linking them to either approval speed or the approval decision. The revolving door between FDA and industry, for example, is well documented.^34,64^ Yet we identified no study testing whether it systematically influenced approval outcomes. This was also true of pre-submission collaborations, congressional lobbying, and political and administrative pressure on approval decisions. Given how often these mechanisms are invoked in policy debates about FDA reform, systematic empirical testing is needed. However, this absence should not be misinterpreted as evidence that factors beyond clinical evidence do not influence the FDA. Such influence is hard to test, and may operate over broader timeframes, for example in shifts to evidentiary standards over time.^25^

### Limitations of evidence and future research

Common use of approval speed (10/15 studies) as a primary outcome may reflect policy developments aimed at speeding up regulatory review times (eg, PDUFA post-1992),^22^ or the fact that speed is a common approach used to evaluate decisions of other regulatory agencies.^47^ Yet while speed matters to Congress and industry, it ignores whether faster approvals necessarily benefit patients.^65,66^ A more population-health-relevant focus would be to evaluate whether approvals are aligned with patient interests. For example, future studies could examine factors associated with the approval of drugs with limited or no evidence of meaningful clinical benefit.

The empirical approaches adopted by authors of the identified studies also warrant comment. All studies relied on observational data, with most identifying associations (eg, between firm characteristics and review times,^45^ media and advocacy salience and review times,^47^ and committee votes and approval decisions.^57^ A smaller number estimated causal effects, exploiting regulatory reforms as natural experiments.^46,48,49^ Quasi-experimental approaches that mimic randomization could help establish the causal impact of factors beyond clinical evidence on approval decisions.

Few studies considered the clinical evidence underpinning approval alongside other tested factors. This is an important limitation, since approval decisions are legitimately shaped by the clinical evidence and broader clinical context such as disease severity, unmet need, availability of alternative treatments, and strength of evidence stemming from use of surrogates, or other trial design characteristics. Without accounting for these, it is difficult to distinguish undue influence from the FDA's legitimate use of discretion. Future work could consider the broader clinical context alongside other factors beyond clinical evidence considered here, enabling a more holistic, patient-outcome-focused study of drug regulatory decision-making.

Factors hypothesized to influence FDA decision-making—political salience, unmet need, and disease advocacy—may vary by disease area, yet several earlier studies did not clearly distinguish between them. This may be a missed opportunity, particularly where external pressures converge in one therapy area and the mechanisms of informal influence may differ. In oncology, for example, high salience, well-organized advocacy and commercial significance coincide with clinical uncertainty arising from the use of surrogate endpoints and other trial design features.^67^ Tibau et al.^55^ documented the FDA's application of flexible evidentiary standards in this area. Elsewhere, patient numbers, salience, and advocacy may be less significant. A disease-specific approach would also allow analysis of multiple FDA decisions along the regulatory lifecycle—from accelerated approval to conversion (to full approval) and withdrawal—while holding clinical context relatively comparable. Together, these points underscore the potential value of interdisciplinary work that combines clinical nuance with rigorous theoretical and empirical methods.

### Limitations of this review

The scope of this review necessarily limits its conclusions. As with any review, the number of studies identified reflects our eligibility criteria; we included only empirical studies in which authors explicitly linked their findings to FDA decision-making. A broader scope, encompassing less explicitly linked work, or alternative outcomes, would have yielded a different, possibly larger, set of eligible studies. Non-empirical or theoretical studies may have proposed useful mechanisms or frameworks not captured here. Some included studies also draw on approval cohorts now decades old, and the intervening years offer scope to retest similar hypotheses on larger, more contemporary datasets. Additionally, studies evaluating outcomes beyond individual approval decisions, such as postmarketing safety events, safety labeling changes, withdrawals, or shifts in evidence standards for approval over time, may together provide a more complete picture of how factors beyond clinical evidence shape FDA decision-making. Nonetheless, given the breadth and interdisciplinary nature of this literature, a focused approach was deemed both necessary and appropriate.

Screening and application of eligibility criteria were primarily conducted by a single reviewer, with a random sample of titles/abstracts and of articles assessed at full-text screening independently screened by a second reviewer. Although use of a single reviewer aligns with PRISMA-ScR guidance, it does increase the risk of missed studies or classification errors. Search results were also limited to English-language publications, which may have excluded relevant studies and introduced language bias.

## Conclusion

Concerns about the influence of factors beyond clinical evidence on FDA drug approval decisions are widespread, yet the empirical basis for these concerns remains limited. The relatively small empirical literature we identified in this scoping review, with mixed findings across the 5 factors examined, likely reflects the inherent difficulty of measuring informal influence empirically, rather than the absence of influence. Future research would benefit from moving beyond approval speed as a primary outcome toward more patient-centered measures, such as whether approved drugs deliver meaningful clinical benefit, to better understand how factors beyond clinical evidence shape the quality of FDA drug approval decisions.

## Supplementary Material

qxag201_Supplementary_Data
