# Supplementary material for: Factors influencing FDA drug approval decisions beyond clinical trial evidence: a scoping review
Source: Health Aff Sch. 2026 Aug 4;4(8):qxag201. doi: 10.1093/haschl/qxag201 (PMC13463631; doi:10.1093/haschl/qxag201)
Supplement: Supplementary file 1 [file qxag201_supplementary_data.zip › Appendix Materials.docx]

**Appendix**

**Appendix A. Systematic search terms**

Medline search (via OVID):

| **Search No.** | **Description** | **Search terms** |
| --- | --- | --- |
| **1** | **Keyword concept #1 FDA** | "United States Food and Drug Administration"/ OR ("Food and Drug Administration" OR FDA* OR USFDA OR "Center for Biologics Evaluation and Research" OR CBER OR "Center for Drug Evaluation and Research" OR CDER OR ((united states OR US OR americ*) adj3 (drug OR medicin* OR pharmaceutical*) adj3 (regulat* OR agenc*))).tw. |
| **2** | **Keyword concept #2 (Drug) Regulatory decisions / actions / outcomes** | ((FDA* or "Food and Drug Administration*") **adj5 (**action* or approv* or inaction* or authori* or behavior* or behaviour* or commitment* or delay* or decid* or decis* or enforc* or "postmarketing requirement*" or "post-marketing requirement*" or (regulat* adj3 (drug* or pharma* or medic* or health*)) or "review time*" or speed or withdraw* or deni* OR deny* or refusal*or recall* OR rul* or verdict* or endorse* or judgement*)).tw. |
| **3** | **Keyword concept #3 Theories of decision making** | (advoca* OR bias* OR bureaucra* OR (regulat* adj3 captur*) OR ((advis* OR oversight) adj2 (committee* OR panel* OR group*)) OR corrupt* OR deliberative OR democra* OR "disease-specific organization*" OR "disease-specific organisation*" OR (economic adj1 (power* or theor* or interest* or influence*)) OR (fail* adj2 regul*) OR ("internal factor*" OR "organization* factor*" OR "organisation* factor*" OR "cultur* factor*" OR "financial factor*" OR "technological factor*" OR "external factor*" OR "economic* factor*" OR "politc* factor*" OR "social factor*" OR "environmental factor*" OR "legal factor*" OR "regulatory factor*" OR "structural factor*" OR "governance factor*" OR "institutional factor*") OR ("internal driver*" OR "organization* driver*" OR "organisation* driver*" OR "cultur* driver*" OR "financial driver*" OR "technological driver*" OR "external driver*" OR "economic* driver*" OR "politc* driver*" OR "social driver*" OR "environmental driver*" OR "legal driver*" OR "regulatory driver*" OR "structural driver*" OR "governance driver*" OR "institutional driver*") OR  ("internal determinant*" OR "organization* determinant*" OR "organisation* determinant*" OR "cultur* determinant*" OR "financial determinant*" OR "technological determinant*" OR "external determinant*" OR "economic* determinant*" OR "politc* determinant*" OR "social determinant*" OR "environmental determinant*" OR "legal determinant*" OR "regulatory determinant*" OR "structural determinant*" OR "governance determinant*" OR "institutional determinant*") OR ("internal influence*" OR "organization* influence*" OR "organisation* influence*" OR "cultur* influence*" OR "financial influence*" OR "technological influence*" OR "external influence*" OR "economic* influence*" OR "politc* influence*" OR "social influence*" OR "environmental influence*" OR "legal influence*" OR "regulatory influence*" OR "structural influence*" OR "governance influence*" OR "institutional influence*") OR  ("internal variable*" OR "organization* variable*" OR "organisation* variable*" OR "cultur* variable*" OR "financial variable*" OR "technological variable*" OR "external variable*" OR "economic* variable*" OR "politc* variable*" OR "social variable*" OR "environmental variable*" OR "legal variable*" OR "regulatory variable*" OR "structural variable*" OR "governance variable*" OR "institutional variable*") OR ("internal condition*" OR "organization* condition*" OR "organisation* condition*" OR "cultur* condition*" OR "financial condition*" OR "technological condition*" OR "external condition*" OR "economic* condition*" OR "politc* condition*" OR "social condition*" OR "environmental condition*" OR "legal condition*" OR "regulatory condition*" OR "structural condition*" OR "governance condition*" OR "institutional condition*") OR ("internal aspect*" OR "organization* aspect*" OR "organisation* aspect*" OR "cultur* aspect*" OR "financial aspect*" OR "technological aspect*" OR "external aspect*" OR "economic* aspect*" OR "politc* aspect*" OR "social aspect*" OR "environmental aspect*" OR "legal aspect*" OR "regulatory aspect*" OR "structural aspect*" OR "governance aspect*" OR "institutional aspect*") OR  ("internal issue*" OR "organization* issue*" OR "organisation* issue*" OR "cultur* issue*" OR "financial issue*" OR "technological issue*" OR "external issue*" OR "economic* issue*" OR "politc* issue*" OR "social issue*" OR "environmental issue*" OR "legal issue*" OR "regulatory issue*" OR "structural issue*" OR "governance issue*" OR "institutional issue*") OR ("internal force*" OR "organization* force*" OR "organisation* force*" OR "cultur* force*" OR "financial force*" OR "technological force*" OR "external force*" OR "economic* force*" OR "politc* force*" OR "social force*" OR "environmental force*" OR "legal force*" OR "regulatory force*" OR "structural force*" OR "governance force*" OR "institutional force*") OR ("internal dynamic*" OR "organization* dynamic*" OR "organisation* dynamic*" OR "cultur* dynamic*" OR "financial dynamic*" OR "technological dynamic*" OR "external dynamic*" OR "economic* dynamic*" OR "politc* dynamic*" OR "social dynamic*" OR "environmental dynamic*" OR "legal dynamic*" OR "regulatory dynamic*" OR "structural dynamic*" OR "governance dynamic*" OR "institutional dynamic*") OR  ("internal pressure*" OR "organization* pressure*" OR "cultur* pressure*" OR "financial pressure*" OR "technological pressure*" OR "external pressure*" OR "economic* pressure*" OR "politc* pressure*" OR "social pressure*" OR "environmental pressure*" OR "legal pressure*" OR "regulatory pressure*" OR "structural pressure*" OR "governance pressure*" OR "institutional pressure*") OR firm* OR "gift-giving" OR impartial* OR implement* OR (fail* adj2 (regul* OR act OR implement*)) OR ((FDA or "Food and Drug Administration*") adj3 independen*) OR ((patient* OR undue OR industr*) adj3 influenc*) OR informal OR "Information* processing" OR "Information* asymmet*" OR institution* OR ((group* OR conflict* OR public* OR patient* OR industr* OR financial OR organised OR special) adj2 interest*) OR (FDA adj3 mandat*) OR media OR "organizational image" OR "organisational image" OR "patient involvement" OR "patient participation" OR "patient representation" OR "patient voice" OR "patient engagement" OR "Patient organization*" OR "patient organisation*" OR "Patient group*" OR Politic* OR Power* OR "press coverage" OR ("Public* interest" OR "Public* accountability" OR "Public* campaign*" OR "Public* controvers*" OR "Public* Involvement" OR "Public* Participation" OR "Public official*") OR Publicity OR rulemaking OR Error* OR Erroneous OR Reputation* OR (FDA* adj5 resource*) OR "Internal resource*" OR (FDA* adj3 fund*) OR "Revolving door" OR Salien* OR Sociopolitic* OR sociopolitic* OR (vote* or voting) ).tw |
| **4** | **1 AND 2 AND 3** | 1 AND 2 AND 3 |
| **5** | **4 And Publication Type (clinical trials)** | limit 4 to (adaptive clinical trial or clinical conference or clinical study or clinical trial, all or clinical trial, phase i or clinical trial, phase ii or clinical trial, phase iii or clinical trial, phase iv or clinical trial, veterinary or clinical trial protocol or clinical trial or controlled clinical trial or pragmatic clinical trial or randomized controlled trial or randomized controlled trial, veterinary) |
| **6** | **4 NOT 5** | 4 NOT 5 |
| **7** | **5 AND >1990 and English Language** | limit 6 to (english language and yr="1990 -Current") |

Scopus search:

| **Search No.** | **Description** | **Search terms** |
| --- | --- | --- |
| **1** | **Keyword concept #1 FDA** | TITLE-ABS(("United States Food and Drug Administration" OR "Food and Drug Administration" OR FDA* OR usfda OR "Center for Biologics Evaluation and Research" OR cber OR "Center for Drug Evaluation and Research" OR cder) OR (("united states" OR us OR americ*) W/5 (drug OR medicin* OR pharmaceutical*) W/3 (regulat* OR agenc*))) |
| **2** | **Keyword concept #2 (Drug) Regulatory decisions / actions / outcomes** | TITLE-ABS ( ( fda* OR "Food and Drug Administration*" ) W/5 ( action* OR approval OR inaction* OR approv* OR authori* OR behavior* OR behaviour* OR commitment* OR delay* OR decid* OR decis* OR enforc* OR ( "postmarketing requirement*" OR "post-marketing requirement*" ) OR ( regulat* W/3 ( drug* OR pharma* OR medic* OR health* ) ) OR "review time*" OR speed OR ( "time to approval*" OR "time to decision*" ) OR withdraw* OR deni* OR deny* OR refusal* OR recall* OR rul* OR verdict* OR endorse* OR judgement*)) |
| **3** | **Keyword concept #3 Theories of decision making** | TITLE-ABS(advoca* OR bias* OR bureaucra* OR (regulat* W/3 captur*) OR ((advis* OR oversight) W/2 (committee* OR panel* OR group*)) OR corrupt* OR deliberative OR democra* OR "disease-specific organization*" OR "disease-specific organisation*" OR (economic W/1 (power* or theor* or interest* or influence*)) OR (fail* W/2 regul*) OR ("internal factor*" OR "organization* factor*" OR "organisation* factor*" OR "cultur* factor*" OR "financial factor*" OR "technological factor*" OR "external factor*" OR "economic* factor*" OR "politc* factor*" OR "social factor*" OR "environmental factor*" OR "legal factor*" OR "regulatory factor*" OR "structural factor*" OR "governance factor*" OR "institutional factor*") OR ("internal driver*" OR "organization* driver*" OR "organisation* driver*" OR "cultur* driver*" OR "financial driver*" OR "technological driver*" OR "external driver*" OR "economic* driver*" OR "politc* driver*" OR "social driver*" OR "environmental driver*" OR "legal driver*" OR "regulatory driver*" OR "structural driver*" OR "governance driver*" OR "institutional driver*") OR ("internal determinant*" OR "organization* determinant*" OR "organisation* determinant*" OR "cultur* determinant*" OR "financial determinant*" OR "technological determinant*" OR "external determinant*" OR "economic* determinant*" OR "politc* determinant*" OR "social determinant*" OR "environmental determinant*" OR "legal determinant*" OR "regulatory determinant*" OR "structural determinant*" OR "governance determinant*" OR "institutional determinant*") OR  ("internal influence*" OR "organization* influence*" OR "organisation* influence*" OR "cultur* influence*" OR "financial influence*" OR "technological influence*" OR "external influence*" OR "economic* influence*" OR "politc* influence*" OR "social influence*" OR "environmental influence*" OR "legal influence*" OR "regulatory influence*" OR "structural influence*" OR "governance influence*" OR "institutional influence*") OR  ("internal variable*" OR "organization* variable*" OR "organisation* variable*" OR "cultur* variable*" OR "financial variable*" OR "technological variable*" OR "external variable*" OR "economic* variable*" OR "politc* variable*" OR "social variable*" OR "environmental variable*" OR "legal variable*" OR "regulatory variable*" OR "structural variable*" OR "governance variable*" OR "institutional variable*") OR ("internal condition*" OR "organization* condition*" OR "organisation* condition*" OR "cultur* condition*" OR "financial condition*" OR "technological condition*" OR "external condition*" OR "economic* condition*" OR "politc* condition*" OR "social condition*" OR "environmental condition*" OR "legal condition*" OR "regulatory condition*" OR "structural condition*" OR "governance condition*" OR "institutional condition*") OR ("internal aspect*" OR "organization* aspect*" OR "organisation* aspect*" OR "cultur* aspect*" OR "financial aspect*" OR "technological aspect*" OR "external aspect*" OR "economic* aspect*" OR "politc* aspect*" OR "social aspect*" OR "environmental aspect*" OR "legal aspect*" OR "regulatory aspect*" OR "structural aspect*" OR "governance aspect*" OR "institutional aspect*") OR ("internal issue*" OR "organization* issue*" OR "organisation* issue*" OR "cultur* issue*" OR "financial issue*" OR "technological issue*" OR "external issue*" OR "economic* issue*" OR "politc* issue*" OR "social issue*" OR "environmental issue*" OR "legal issue*" OR "regulatory issue*" OR "structural issue*" OR "governance issue*" OR "institutional issue*") OR ("internal force*" OR "organization* force*" OR "organisation* force*" OR "cultur* force*" OR "financial force*" OR "technological force*" OR "external force*" OR "economic* force*" OR "politc* force*" OR "social force*" OR "environmental force*" OR "legal force*" OR "regulatory force*" OR "structural force*" OR "governance force*" OR "institutional force*") OR ("internal dynamic*" OR "organization* dynamic*" OR "organisation* dynamic*" OR "cultur* dynamic*" OR "financial dynamic*" OR "technological dynamic*" OR "external dynamic*" OR "economic* dynamic*" OR "politc* dynamic*" OR "social dynamic*" OR "environmental dynamic*" OR "legal dynamic*" OR "regulatory dynamic*" OR "structural dynamic*" OR "governance dynamic*" OR "institutional dynamic*") OR ("internal pressure*" OR "organization* pressure*" OR "cultur* pressure*" OR "financial pressure*" OR "technological pressure*" OR "external pressure*" OR "economic* pressure*" OR "politc* pressure*" OR "social pressure*" OR "environmental pressure*" OR "legal pressure*" OR "regulatory pressure*" OR "structural pressure*" OR "governance pressure*" OR "institutional pressure*") OR firm* OR "gift-giving" OR impartial* OR implement* OR (fail* W/2 (regul* OR act OR implement*)) OR ((FDA or "Food and Drug Administration*") W/3 independen*) OR ((patient* OR undue OR industr*) W/3 influenc*) OR informal OR "Information* processing" OR "Information* asymmet*" OR institution* OR ((group* OR conflict* OR public* OR patient* OR industr* OR financial OR organised OR special) W/2 interest*) OR (FDA W/3 mandat*) OR media OR "organizational image" OR "organisational image" OR "patient involvement" OR "patient participation" OR "patient representation" OR "patient voice" OR "patient engagement" OR "Patient organization*" OR "patient organisation*" OR "Patient group*" OR Politic* OR Power* OR "press coverage" OR ("Public* interest" OR "Public* accountability" OR "Public* campaign*" OR "Public* controvers*" OR "Public* Involvement" OR "Public* Participation" OR "Public official*") OR Publicity OR rulemaking OR Error* OR Erroneous OR Reputation* OR (FDA* W/5 resource*) OR "Internal resource*" OR (FDA* W/3 fund*) OR "Revolving door" OR Salien* OR Sociopolitic* OR sociopolitic* OR (vote* or voting)) |
| **4** | **1 AND 2 AND 3** | 1 AND 2 AND 3 |
| **5** | **4 AND Remove clinical trials** | 4 AND NOT TITLE("clinical trial" OR "randomized controlled trial" OR "phase I" OR "phase 1" OR "phase ii" OR "phase 2" OR "phase iii" or "phase 3") |
| **6** | **5 AND >1990 and English Langauge  (full search terms)** | LANGUAGE ( english ) PUBYEAR > 1989 TITLE-ABS ( ( "United States Food and Drug Administration" OR "Food and Drug Administration" OR fda* OR usfda OR "Center for Biologics Evaluation and Research" OR cber OR "Center for Drug Evaluation and Research" OR cder ) OR ( ( "united states" OR us OR americ* ) W/5 ( drug OR medicin* OR pharmaceutical* ) W/3 ( regulat* OR agenc* ) ) ) AND TITLE-ABS ( ( fda* OR "Food and Drug Administration*" ) W/5 ( action* OR approval OR inaction* OR approv* OR authori* OR behavior* OR behaviour* OR commitment* OR delay* OR decid* OR decis* OR enforc* OR ( "postmarketing requirement*" OR "post-marketing requirement*" ) OR ( regulat* W/3 ( drug* OR pharma* OR medic* OR health* ) ) OR "review time*" OR speed OR ( "time to approval*" OR "time to decision*" ) OR withdraw* OR deni* OR deny* OR refusal* OR recall* OR rul* OR verdict* OR endorse* OR judgement* ) ) AND TITLE-ABS ( advoca* OR bias* OR bureaucra* OR ( regulat* W/3 captur* ) OR ( ( advis* OR oversight ) W/2 ( committee* OR panel* OR group* ) ) OR corrupt* OR deliberative OR democra* OR "disease-specific organization*" OR "disease-specific organisation*" OR ( economic W/1 ( power* OR theor* OR interest* OR influence* ) ) OR ( fail* W/2 regul* ) OR ( "internal factor*" OR "organization* factor*" OR "organisation* factor*" OR "cultur* factor*" OR "financial factor*" OR "technological factor*" OR "external factor*" OR "economic* factor*" OR "politc* factor*" OR "social factor*" OR "environmental factor*" OR "legal factor*" OR "regulatory factor*" OR "structural factor*" OR "governance factor*" OR "institutional factor*" ) OR ( "internal driver*" OR "organization* driver*" OR "organisation* driver*" OR "cultur* driver*" OR "financial driver*" OR "technological driver*" OR "external driver*" OR "economic* driver*" OR "politc* driver*" OR "social driver*" OR "environmental driver*" OR "legal driver*" OR "regulatory driver*" OR "structural driver*" OR "governance driver*" OR "institutional driver*" ) OR ( "internal determinant*" OR "organization* determinant*" OR "organisation* determinant*" OR "cultur* determinant*" OR "financial determinant*" OR "technological determinant*" OR "external determinant*" OR "economic* determinant*" OR "politc* determinant*" OR "social determinant*" OR "environmental determinant*" OR "legal determinant*" OR "regulatory determinant*" OR "structural determinant*" OR "governance determinant*" OR "institutional determinant*" ) OR ( "internal influence*" OR "organization* influence*" OR "organisation* influence*" OR "cultur* influence*" OR "financial influence*" OR "technological influence*" OR "external influence*" OR "economic* influence*" OR "politc* influence*" OR "social influence*" OR "environmental influence*" OR "legal influence*" OR "regulatory influence*" OR "structural influence*" OR "governance influence*" OR "institutional influence*" ) OR ( "internal variable*" OR "organization* variable*" OR "organisation* variable*" OR "cultur* variable*" OR "financial variable*" OR "technological variable*" OR "external variable*" OR "economic* variable*" OR "politc* variable*" OR "social variable*" OR "environmental variable*" OR "legal variable*" OR "regulatory variable*" OR "structural variable*" OR "governance variable*" OR "institutional variable*" ) OR ( "internal condition*" OR "organization* condition*" OR "organisation* condition*" OR "cultur* condition*" OR "financial condition*" OR "technological condition*" OR "external condition*" OR "economic* condition*" OR "politc* condition*" OR "social condition*" OR "environmental condition*" OR "legal condition*" OR "regulatory condition*" OR "structural condition*" OR "governance condition*" OR "institutional condition*" ) OR ( "internal aspect*" OR "organization* aspect*" OR "organisation* aspect*" OR "cultur* aspect*" OR "financial aspect*" OR "technological aspect*" OR "external aspect*" OR "economic* aspect*" OR "politc* aspect*" OR "social aspect*" OR "environmental aspect*" OR "legal aspect*" OR "regulatory aspect*" OR "structural aspect*" OR "governance aspect*" OR "institutional aspect*" ) OR ( "internal issue*" OR "organization* issue*" OR "organisation* issue*" OR "cultur* issue*" OR "financial issue*" OR "technological issue*" OR "external issue*" OR "economic* issue*" OR "politc* issue*" OR "social issue*" OR "environmental issue*" OR "legal issue*" OR "regulatory issue*" OR "structural issue*" OR "governance issue*" OR "institutional issue*" ) OR ( "internal force*" OR "organization* force*" OR "organisation* force*" OR "cultur* force*" OR "financial force*" OR "technological force*" OR "external force*" OR "economic* force*" OR "politc* force*" OR "social force*" OR "environmental force*" OR "legal force*" OR "regulatory force*" OR "structural force*" OR "governance force*" OR "institutional force*" ) OR ( "internal dynamic*" OR "organization* dynamic*" OR "organisation* dynamic*" OR "cultur* dynamic*" OR "financial dynamic*" OR "technological dynamic*" OR "external dynamic*" OR "economic* dynamic*" OR "politc* dynamic*" OR "social dynamic*" OR "environmental dynamic*" OR "legal dynamic*" OR "regulatory dynamic*" OR "structural dynamic*" OR "governance dynamic*" OR "institutional dynamic*" ) OR ( "internal pressure*" OR "organization* pressure*" OR "cultur* pressure*" OR "financial pressure*" OR "technological pressure*" OR "external pressure*" OR "economic* pressure*" OR "politc* pressure*" OR "social pressure*" OR "environmental pressure*" OR "legal pressure*" OR "regulatory pressure*" OR "structural pressure*" OR "governance pressure*" OR "institutional pressure*" ) OR firm* OR "gift-giving" OR impartial* OR implement* OR ( fail* W/2 ( regul* OR act OR implement* ) ) OR ( ( fda OR "Food and Drug Administration*" ) W/3 independen* ) OR ( ( patient* OR undue OR industr* ) W/3 influenc* ) OR informal OR "Information* processing" OR "Information* asymmet*" OR institution* OR ( ( group* OR conflict* OR public* OR patient* OR industr* OR financial OR organised OR special ) W/2 interest* ) OR ( fda W/3 mandat* ) OR media OR "organizational image" OR "organisational image" OR "patient involvement" OR "patient participation" OR "patient representation" OR "patient voice" OR "patient engagement" OR "Patient organization*" OR "patient organisation*" OR "Patient group*" OR politic* OR power* OR "press coverage" OR ( "Public* interest" OR "Public* accountability" OR "Public* campaign*" OR "Public* controvers*" OR "Public* Involvement" OR "Public* Participation" OR "Public official*" ) OR publicity OR rulemaking OR error* OR erroneous OR reputation* OR ( fda* W/5 resource* ) OR "Internal resource*" OR ( fda* W/3 fund* ) OR "Revolving door" OR salien* OR sociopolitic* OR sociopolitic* OR ( vote* OR voting ) ) AND NOT TITLE ( "clinical trial" OR "randomized controlled trial" OR "randomised controlled trial" OR "phase I" OR "phase 1" OR "phase ii" OR "phase 2" OR "phase iii" OR "phase 3" ) |

Appendix B. Examples of papers out of scope of this review

| **Author, Year, Title** | **Study discipline** | **Brief study overview** | **Key findings** | **Notes/ reason for exclusion** |
| --- | --- | --- | --- | --- |
| **Study focus out of scope** (not on the FDA) | | | | |
| Yeoh and Roth (1999)  An empirical analysis of sustained advantage in the U.S. pharmaceutical industry: Impact of firm resources and capabilities. | Management / political science | The study evaluates how do firm resources and capabilities influence their sustained competitive advantage in the pharmaceutical industry, as measured by therapeutic differentiation and regulatory success. | - R&D expenditures influenced sustained advantage  - Therapeutic market focus and other factors play a part in success  - Internal R&D and scale-related advantages of firms linked to FDA approval success | This study does not focus on FDA decision making, but rather firms’ capabilities and characteristics that drive commercial success. |
| **Study type out of scope** (not empirical study) | | | | |
| Gyawali and Goldstein (2018). The US Food and Drug Administration’s Approval of Adjuvant Sunitinib for Renal Cell Cancer: A Case of Regulatory Capture? | Health policy | The study critiques FDA’s 2017 approval of adjuvant sutinib for renal cell carcinoma, citing weak clinical evidence and potential industry influence. | - Sutinib was approved despite demonstration of lack of clinical benefit in the indicated population. | This is a descriptive study focused on a single FDA drug approval. It does not empirically evaluate how informal or institutional factors influence FDA approval decisions. |
| **Outcome variables out of scope** (not approval decisions) | | | | |
| Gusmano, M. K. (2013). FDA Decisions and Public Deliberation: Challenges and Opportunities. | Public administration, political science | The study examines how and to what extent public and patient participation can and should be integrated into FDA’s drug approval and regulatory decision-making processes. | - The FDA's current approach to public participation is limited, passive, and often symbolic.  - Greater use of structured deliberative forums could enhance public trust and improve how the FDA assesses risk and benefit. | This study focusses on public and patient involvement across the full FDA regulatory process, rather than drug approval decisions. |
| Herder (2019). Pharmaceutical Drugs of Uncertain Value, Lifecycle Regulation at the US Food and Drug Administration, and Institutional Incumbency. | Health law and policy, regulatory science, political science | The study investigates legal, institutional, and political factors that shape the FDA’s use and enforcement of postmarketing requirements (PMRs). | - FDA struggles to enforce PMRs due to limited resources, unclear legal authority, and political pressure against withdrawing approvals.  - Officials maintain lifecycle regulation despite its flaws, reflecting a defensive stance to protect institutional power or “institutional incumbency”. | This study focusses on FDA’s enforcement of post-marketing requirements, rather than drug approval decisions. |
| Pham-Kanter (2014). Revisiting financial conflicts of interest in FDA advisory committees. | Health policy, public administration | The study examines whether financial conflicts of interest influence FDA advisory committee members votes on drug approval questions. | - Members with exclusive financial ties to the sponsor were significantly more likely to vote in favor of the sponsor. Advisory board ties had the strongest bias. | The study does not evaluate FDA approval decisions as the outcome. |
| **Explanatory variables out of scope** (not informal/ institutional factors) | | | | |
| Thomas (1990). Regulation and Firm Size: FDA Impacts on Innovation | Health economics | The study investigates how FDA regulation influences drug innovation and how these effects vary by firm size. | Increased cost and time of drug development (since 1962 amendments) may have unintentionally shifted innovative capacity toward large firms. | The study focusses on the impact of FDA on innovation, rather than informal or institutional factors that influence FDA decision-making. |
| Dranove & Meltzer (1994). Do Important Drugs Reach the Market Sooner? | Health economics, Innovation | Examines whether drugs perceived as scientifically or commercially important are approved faster by the FDA. | - More important drugs were approved significantly faster by the FDA. | The study does not analyse informal or institutional factors that influence FDA decision-making. |
| DiMasi et al. (1997)  Initiatives to Speed New Drug Development and Regulatory Review- The Impact of FDA-Sponsor Conferences. | Health economics, regulatory science | The study evaluated whether formal FDA-sponsor conferences (meetings) reduce new drug development reduce review times. | Agency familiarity with data on individual drugs before, and during, formal review leads to shorter review times. | The study does not analyse informal or institutional factors that influence FDA decision-making. |
| Kaitin and DiMasi (2000). Measuring the Pace of New Drug Development in the User Fee ERA. | Regulatory science, Innovation | The study measures the speed of new drug development and regulatory review before and after the implementation of the Prescription Drug User Fee Act (PDUFA), and evaluates trends in development and approval timelines. | - FDA review times decreased significantly post-PDUF  - Total development time did not decline and may have increased slightly. | The study does not analyse informal or institutional factors that influence FDA decision-making. |
| Janiaud et al (2021). FDA Reasoning in Approval Decisions When Efficacy Evidence Is Borderline. | Health policy, regulatory science | The study aims to evaluate FDA decision-making behind drugs that were initially rejected for approval. | - The FDA’s threshold for "substantial evidence" was applied inconsistently, often decisions lacked citation of precedent.  - The FDA has no systematic method or culture of referencing past decisions, undermining consistency and institutional memory. | While the study is empirical and identifies "evidential weaknesses" and other key "characteristics" influencing FDA decisions, the study does not systematically test or evaluate institutional or organizational factors (i.e., those beyond clinical evidence) as explanatory variables. |

**Notes:** Table illustrative only (non-exhaustive)
